# A Comparative Study of Novel Fibrosis Index and Other Non-invasive Serum Indices for Predicting Fibrosis in Patients of Chronic Liver Disease

**DOI:** 10.7759/cureus.63658

**Published:** 2024-07-02

**Authors:** Kaustubh Singh, Mahak Lamba, Vivek Kumar, Pahul Ahuja, K. K Gupta, Himanshu Reddy, Ajay Patwa, Sumit Rungta, Sudhir Verma

**Affiliations:** 1 Internal Medicine, King George's Medical University, Lucknow, IND; 2 Gastroenterology, King George's Medical University, Lucknow, IND; 3 Internal Medicine, King George’s Medical University, Lucknow, IND

**Keywords:** cirrhosis, hepatic fibrosis, transient elastography (fibroscan), chronic liver disease, novel fibrosis index

## Abstract

Introduction

Chronic liver disease progression leads to liver fibrosis/cirrhosis. Transient Elastography is used for staging liver fibrosis but ascites, obesity, and operator experience limit its applicability. In this study, we compared various non-invasive serum indices in predicting fibrosis in chronic liver disease patients.

Materials and methods

A total of 142 cases of confirmed Chronic Liver Disease were included. Quantitative determination of liver stiffness by Transient Elastography and relevant blood investigations was done. We compared the liver stiffness measurement by Transient Elastography and fibrosis indices, i.e., Aspartate Transaminase (AST) to Alanine Transaminase (ALT) Ratio (AAR), AST to Platelet Ratio Index (APRI), Fibrosis Index (FI), Fibrosis-4 (FIB-4) Index, Age-Platelet Index (API), Pohl score, and Fibrosis Cirrhosis Index (FCI) with Novel Fibrosis Index (NFI), to predict liver fibrosis stages.

Results

The optimum cutoff of NFI for the F4 stage was ≥ 6670 with a sensitivity of 75.8% and specificity of 81.8%, for the F3 stage was ≥ 2112 with a sensitivity of 63.6% and specificity of 72.7%, and for the F2 stage was ≥ 1334 with a sensitivity of 100% and specificity of 56.3%. The NFI had the maximum area under the curve compared to other indices in predicting fibrosis stages.

Conclusion

The Novel Fibrosis Index was the best in predicting fibrosis stages in Chronic Liver Disease patients, with good performance in predicting the F4 stage.

## Introduction

Chronic liver disease is a progressive deterioration of liver functions for more than six months. It is a continuous process of inflammation, destruction, and regeneration of liver parenchyma, which leads to fibrosis [1].

Non-invasive assessment of liver fibrosis has experienced explosive growth in recent years and a wide spectrum of non-invasive methods ranging from serum assays to imaging techniques have been developed [2]. Several non-invasive markers have been developed that are useful supplements to assess stages of fibrosis like Aspartate Transaminase (AST) to Alanine Transaminase (ALT) Ratio (AAR), AST to Platelet Ratio Index (APRI), Fibrosis Index (FI), Fibrosis-4 (FIB-4) Index, Age-Platelet Index (API), Pohl score, Fibrosis Cirrhosis Index (FCI), and radiological markers including Magnetic Resonance Imaging (MRI) and Transient Elastography [3].

Failure of liver stiffness measurement by Transient Elastography (Fibroscan) and unreliable results occur in 5% and 15% of patients, respectively, mainly due to obesity. It is not useful in patients with ascites [4]. Another disadvantage associated with Fibroscan is its cost and unavailability in resource-poor and remote settings.

To overcome this challenge, the Novel Fibrosis Index (NFI) was developed by Husain AR et al [3]. NFI is a newly developed noninvasive serum index for predicting liver fibrosis. The index was developed in the year 2019. This index uses Serum Bilirubin, Serum Alkaline Phosphatase, Platelet Count, and Serum Albumin as variables, where n=2000 and ‘n’ is a constant that is introduced to accommodate measurement in small values which are more convenient to use [3].

In the study done by Husain AR et al [3], NFI was found to have more sensitivity and specificity in predicting the F4 fibrosis stage than any other available non-invasive serum indices. It also predicted the F3 stage with considerable sensitivity and specificity.

There is a paucity of literature on the applicability of NFI in predicting liver fibrosis. Therefore, this study was carried out to validate and compare NFI with other available non-invasive serum indices.

This article was previously presented as an abstract in APICON 2022.

## Materials and methods

The cross-sectional observational study was conducted in the Department of Medicine, King George’s Medical University (KGMU), Lucknow. All the patients were included in the study after getting ethical clearance from the Institutional Ethics Committee, King George’s Medical University (KGMU), Lucknow (Ref. code: 103rd ECM II B-Thesis/P21). All those patients (OPD/IPD) of age > 18 years who had evidence of liver dysfunction for >6 months in the form of either ultrasonographic abnormality (hepatomegaly/irregular margins/coarse echotexture) or elevated liver enzymes were included in the study. The study excluded patients having hyperacute/acute/subacute liver failure (jaundice and coagulopathy with onset of encephalopathy within 12 weeks of onset of jaundice [5]), acute viral hepatitis, acute on chronic liver failure (based on diagnostic criteria laid down by European Association for the Study of the Liver [6]), hepatocellular carcinoma and pregnant women.

A total of 142 patients with chronic liver disease were taken and determination of liver stiffness using Transient Elastography was done. Relevant blood investigations (liver function tests and complete blood count) were done. The fibrosis stages of patients were determined from liver stiffness by fibro scan using the Meta-Analysis of Histological Data in Viral Hepatitis (METAVIR) System. Ziol transient elastography breaking points for the staging of fibrosis according to the METAVIR System of fibrosis were used (Table 1).

**Table 1:** Liver stiffness cut-off values for different stages of fibrosis kPa : kilopascals Based on Ziol et al. [7]

Liver Stiffness (in kPa)	Stage of Fibrosis
< 8.8	F0 - F1
8.9 – 9.6	F2
9.7 – 14.6	F3
>14.6	F4

The patients were assessed for NFI. The result of the fibroscan score and NFI were correlated. Also, other non-invasive serum indices for predicting liver fibrosis, i.e., AAR, APRI, FI, FIB-4, API, Pohl score, and FCI, were calculated and compared with NFI for predicting various liver fibrosis stages. The formulae for these non-invasive indices are mentioned in Table 2.

**Table 2:** Non-invasive serum indices of liver fibrosis and their formulae mg/dl : milligrams/deciliter, ALP : Alkaline Phosphatase,  PLT: Platelet Count, IU/L : International Units/Liter, S.Alb : Serum Albumin, gm/dL : grams/deciliter Based on Hussain et al. [3]

Non invasive indices	Formulae
Novel Fibrosis Index (NFI)	NFI = {total bilirubin (mg/dL)×(ALP (IU/L))^2^/PLT (lakhs/mm^3^) × (S.Alb (gm/dL))^2^} - n, where n=2000 and ‘n’ is constant.
Aspartate Transaminase (AST) to Alanine Transaminase (ALT) ratio (AAR)	AAR = AST (IU/L) / ALT (IU/L)
AST to Platelet Ratio Index (APRI)	APRI = {{AST (IU/L) / AST upper limit of normal (IU/L)}×100}/ PLT (10^9^/L)
Fibrosis Index (FI)	FI = 8.0 - (0.01 × PLT (10^9^/L)) − S. Alb (gm/dL)
Fibrosis-4 (FIB-4)	FIB-4 = {age (in years) × AST (IU/L)} / {PLT (10^9^/L) × ALT (IU/L)^1/2^}
Fibrosis Cirrhosis Index (FCI)	FCI = (ALP (IU/L) × total bilirubin (mg/dL))/ (S.Alb (gm/dL) × PLT(10^9^/L))
Age-Platelet Index (API)	API = Age/Platelet Index, where Age (years)<30 = 0; 30-39 = 1; 40-49 = 2; 50-59 = 3; 60-69 = 4; ≥70 = 5 and PLT (10^9^/L): ≥225 = 0; 200-224 = 1; 175-199 = 2; 150-174 = 3; 125-149 = 4; <125 = 5. It ranges from 0 to 10, where 0-2 = no or minimal fibrosis, 3-5 = mild fibrosis with few septa formation, and ≥6 = bridging fibrosis to cirrhosis and/or moderate-to-severe necro-inflammatory lesions.
Pohl Score	AST (IU/L) : ALT (IU/L) : PLT(10 ^9^/L) If AST/ALT < 1 and PLT > 150 × 10^9^/L, then excludes marked fibrosis

ROC (Receiver Operating Characteristic) curves were performed and the area under curves was used to collate and infer the diagnostic accuracies of the serum fibrosis indexes along with their cut-off points, sensitivities, and specificities. The level of statistical significance was set as p-value <0.05. In ROC curve analysis, an area of 1 represents a perfect test; an area of 0.5 represents a worthless test. A rough guide for classifying the accuracy of a diagnostic test is the traditional academic point system using AUC (Area Under Curve) as mentioned in Table 3 [8]:

**Table 3:** A guide for classifying the accuracy of a diagnostic test by AUC AUC : Area Under Curve

AUC Range	Classification
0.9 – 1.0	Excellent
0.8 – 0.9	Good
0.7 – 0.8	Fair
0.6 – 0.7	Poor
0.5 – 0.6	Fail

## Results

A total of 142 patients with chronic liver disease were enrolled in the study. The age of patients ranged from 18 to 82 years. The mean age of patients was 45 ± 14.25 years. The majority of patients were males (64.8%) and >40 years of age. Relevant data of routine blood investigations of patients can be seen in Table 4. In the present study, hepatitis virus infection was the most common etiology (47%) (Hepatitis B virus-related 29.3%, Hepatitis C virus-related 23.3%) followed by alcoholism (22%). There were only 31% of cases affected by other etiologies out of which 29% of etiologies were being still investigated. Maximum number of patients belonged to the F4 fibrosis stage (77.4%) followed by F0-F1(12%), F3(8.5%) and F2 (2.1%).

**Table 4:** Descriptive summary of hematological and biochemical parameters AST: Aspartate Aminotransferase; ALT: Alanine Aminotransferase; ALP: Alkaline Phosphatase; INR: International Normalized Ratio

Variables	Minimum	Maximum	Mean	Standard deviation
Hemoglobin (grams/deciliter)	3.30	16.5	9.1	2.6
Total Leucocyte Count (cells/millimeter^3^)	600	31400	7461.4	4986.1
Random Blood Sugar (milligrams/deciliter)	90	120	105.1	8.8
Mean Corpuscular Volume (femtoliter)	66.00	137.0	87.7	11.1
Mean Corpuscular Hemoglobin (picogram)	18.00	38.0	29.3	3.8
Platelet Count (lakh/millimeter^3^)	0.20	4.50	1.1	0.7
Serum Bilirubin Total (milligrams/deciliter)	0.27	29.00	2.8	4.1
Serum Bilirubin Direct (milligrams/deciliter)	0.12	19.00	1.6	2.5
AST (International Units/Liter)	15.00	585.00	88.8	93.5
ALT (International Units/Liter)	9.00	368.00	54.2	51.7
ALP (International Units/Liter)	85.00	1604.00	326.9	214.8
Serum Protein (grams/deciliter)	3.40	9.80	6.4	1.1
Serum Albumin (grams/deciliter)	1.20	5.30	3.0	0.7
Serum Urea (milligrams/deciliter)	8.00	201.00	45.6	32.1
Serum Creatinine (milligrams/deciliter)	0.20	4.70	1.1	0.6
INR (International Normalized Ratio)	1.2	3.2	1.6	0.2

The range of NFI was between 1781.56 and 740569.73 with a mean value of 55161.3 ± 105655.9. The mean values of other non-invasive markers are mentioned in Table 5. The range of Liver Stiffness Score on Fibroscan was between 2.6 kPa and 75 kPa. The mean Liver Stiffness Score on Fibroscan was 40.08 ±23.4 kPa (Table 5).

**Table 5:** Distribution of serum biomarker indices for liver fibrosis and liver stiffness score on fibroscan

Variables	Minimum	Maximum	Mean	Standard deviation
NFI (Novel Fibrosis Index)	1781.56	740569.73	55161.3	105655.9
AAR (Aspartate Transaminase (AST) to Alanine Transaminase (ALT) ratio)	0.54	9.75	1.8	1.0
APRI (AST to Platelet Ratio Index)	0.14	11.21	2.3	1.9
FI (Fibrosis Index)	0.10	5.91	3.9	1.0
FIB-4 (Fibrosis-4)	0.29	32.29	7.0	6.7
FCI (Fibrosis Cirrhosis Index)	0.07	36.47	4.0	6.3
API (Age-Platelet Index)	0.0	3.0	0.5	0.4
Liver Stiffness by Fibroscan (in kilopascals)	2.6	75	40.08	23.4

There was a positive correlation of Liver Stiffness Score on Fibroscan with all biomarker indices. There is a positive significant correlation between Liver Stiffness Score on Fibroscan with NFI (p < 0.05), AAR (p < 0.01), FI (p < 0.01), and FCI (p < 0.01).

The optimal cutoff of the F4 stage for NFI was ≥ 6670 with a sensitivity of 75.8% and specificity of 81.8%. NFI had the maximum Area Under the Receiver Operating Characteristic curve (AUROC) (0.848). The most sensitive index for the F4 stage was the Pohl score (98.9%). The most specific index for the F4 stage was NFI and FCI (81.8%) (Table 6).

**Table 6:** Optimum cut-off for the F4 stage for various indices AUROC : Area Under Receiver Operating Characteristic curve

Parameter	Optimum cut off for F4	Sensitivity	Specificity	AUROC
NFI (Novel Fibrosis Index)	≥6670	75.8	81.8	0.848
AAR (Aspartate Transaminase (AST) to Alanine Transaminase (ALT) ratio)	≥1.32	73.6	54.5	0.665
FI (Fibrosis Index )	≥3.69	73.6	68.2	0.747
FIB-4 (Fibrosis-4)	≥4.19	59.3	77.3	0.662
FCI (Fibrosis Cirrhosis Index)	≥0.97	80.2	81.8	0.836
Pohl Score	≥1	98.9	18.2	0.585
API (Age-Platelet Index)	≥0.55	50.9	67.7	0.580
APRI (AST to Platelet Ratio Index)	≥0.65	94.5	29	0.618

**Figure 1:**
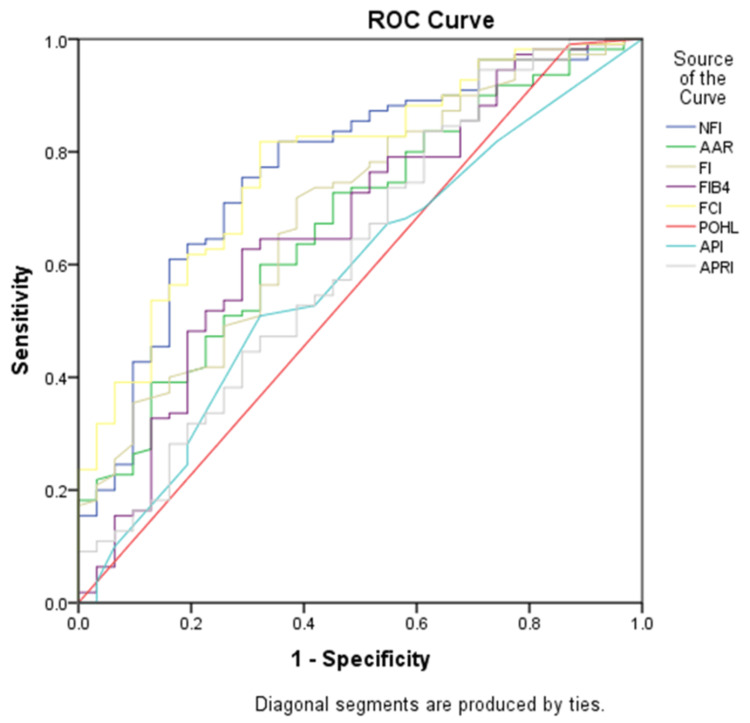
ROC curve analysis for the F4 stage ROC: Receiver Operating Characteristic; NFI: Novel Fibrosis Index; AAR: Aspartate Transaminase (AST) to Alanine Transaminase (ALT) ratio; FI: Fibrosis Index; FIB-4: Fibrosis-4; FCI: Fibrosis Cirrhosis Index; POHL: Pohl Score; API: Age-Platelet Index; APRI: AST to Platelet Ratio Index

The maximum AUC (Area Under Curve) for the F4 stage was for NFI (0.848) and the least AUC was for API (0.580). API (AUC 0.580) and Pohl score (AUC 0.585) failed to predict the F4 stage (Figure 1).

The optimal cutoff of the F3 stage for NFI was ≥ 2112 with a sensitivity of 63.6% and specificity of 72.7%. The most sensitive index for the F3 stage was AAR, FI, and FIB-4 (90.9%). The most specific index for the F3 stage was NFI (72.7%) (Table 7).

**Table 7:** Optimum cut-off for the F3 stage for various indices AUROC : Area Under Receiver Operating Characteristic curve

Parameter	Optimum cut off for F3	Sensitivity	Specificity	AUROC
NFI (Novel Fibrosis Index)	≥2112	63.6	72.7	0.669
AAR (Aspartate Transaminase (AST) to Alanine Transaminase (ALT) ratio)	≥0.93	90.9	45.5	0.636
FI (Fibrosis Index)	≥3.15	90.9	63.6	0.616
FIB-4 (Fibrosis-4)	≥2.39	90.9	45.5	0.603
FCI (Fibrosis Cirrhosis Index)	≥0.45	72.7	54.5	0.603
API (Age-Platelet Index)	≥0.10	83.3	31.6	0.526
APRI (AST to Platelet Ratio Index)	≥1.24	83.3	63.2	0.660

**Figure 2:**
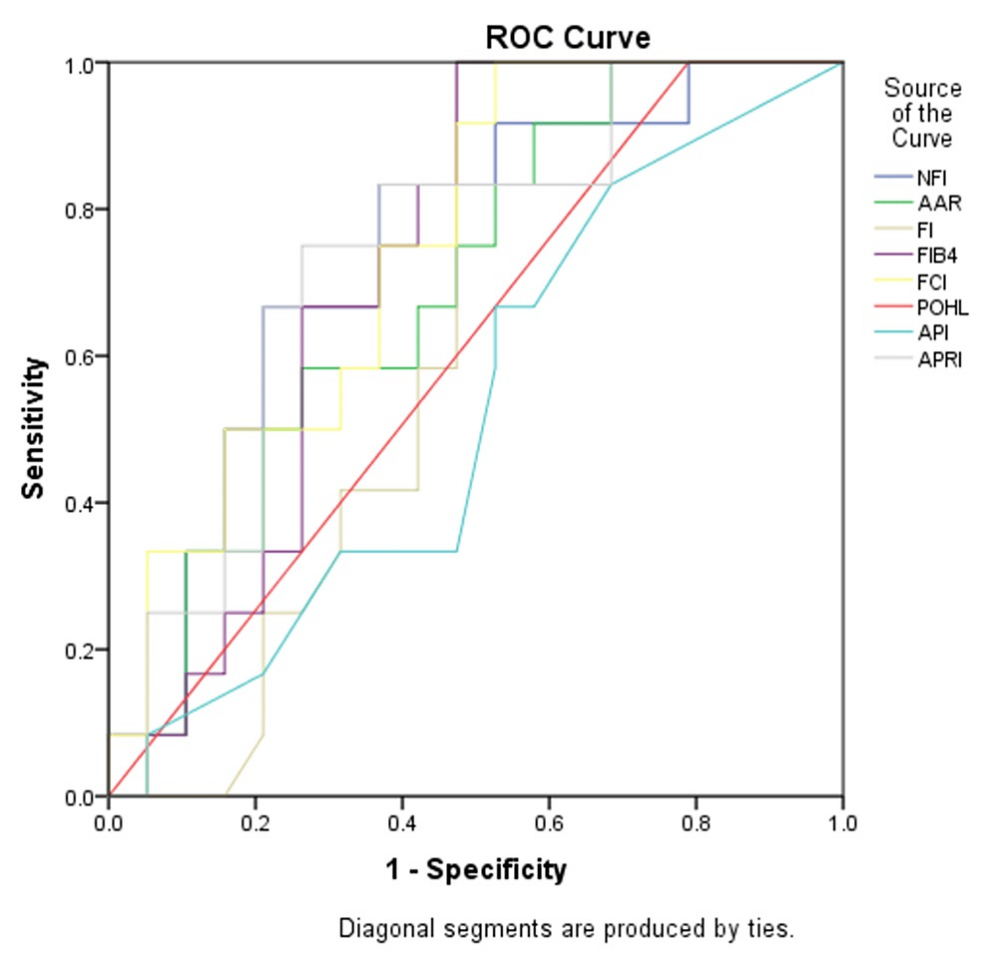
ROC curve analysis for the F3 stage ROC: Receiver Operating Characteristic; NFI: Novel Fibrosis Index; AAR: Aspartate Transaminase (AST) to Alanine Transaminase (ALT) ratio; FI: Fibrosis Index; FIB-4: Fibrosis-4; FCI: Fibrosis Cirrhosis Index; POHL: Pohl Score; API: Age-Platelet Index; APRI: AST to Platelet Ratio Index

The maximum AUC for the F3 stage was for NFI (0.669) and the least AUC was for API (0.526). Hence, among all the non-invasive markers, NFI is the best index to predict the F3 stage of liver fibrosis (Figure 2).

The optimal cutoff of the F2 stage for NFI was ≥1334 with a sensitivity of 100% and specificity of 56.3%. The Fibrosis Cirrhosis Index also had 100% sensitivity for the F2 stage. The Fibrosis Index had the maximum specificity (93.8%) for the F2 stage but very low sensitivity (33.3%) (Table 8).

**Table 8:** Optimum cutoff for the F2 stage for various indices AUROC : Area Under Receiver Operating Characteristic curve

Parameter	Optimum cut off for F2	Sensitivity	Specificity	AUROC
NFI (Novel Fibrosis Index)	≥1334	100	56.3	0.688
AAR (Aspartate Transaminase (AST) to Alanine Transaminase (ALT) ratio)	≥0.53	33.3	81.3	0.354
FI (Fibrosis Index)	≥1.75	33.3	93.8	0.313
FIB-4 (Fibrosis-4)	≥1.55	66.7	43.8	0.396
FCI (Fibrosis Cirrhosis Index)	≥0.21	100	37.5	0.500
API (Age-Platelet Index)	≥0.05	66.7	87.5	0.498
APRI (AST to Platelet Ratio Index)	≥0.80	33.3	75.0	0.292

**Figure 3:**
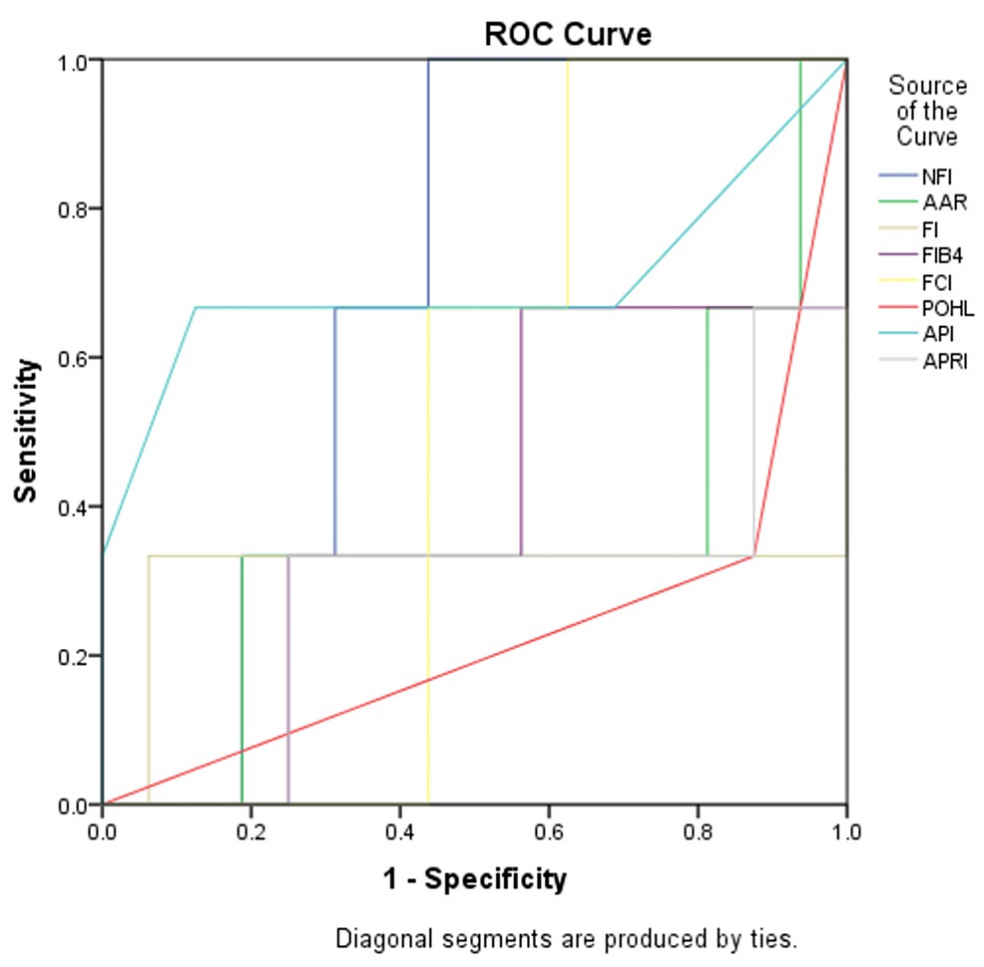
ROC curve analysis for the F2 stage ROC: Receiver Operating Characteristic; NFI: Novel Fibrosis Index; AAR: Aspartate Transaminase (AST) to Alanine Transaminase (ALT) ratio; FI: Fibrosis Index; FIB-4: Fibrosis-4; FCI: Fibrosis Cirrhosis Index; POHL: Pohl Score; API: Age-Platelet Index; APRI: AST to Platelet Ratio Index

The maximum AUC for the F2 stage was for NFI (0.688) and the least AUC was for APRI (0.292). Hence, among all the non-invasive markers, NFI is the best index to predict the F2 stage of liver fibrosis (Figure 3).

## Discussion

Liver cirrhosis is the end-stage of chronic liver disease. Interestingly, more than two-thirds of the patients with cirrhosis are unaware even of having liver disease [9]. For a better prognosis, it is essential to assess the severity of the disease using reliable and cost-effective methods that are able to provide results in the shortest possible time. Liver biopsy has been advised as a gold standard to evaluate the fibrosis stage, yet it is invasive. A number of non-invasive markers have been developed that are useful supplements to assess stages of fibrosis like AAR, APRI, FI, FIB-4, API, Pohl score, FCI, NFI, and radiological markers including MRI and transient elastography. A new index called the Novel Fibrosis Index (NFI) was developed by Husain et al in the year 2019 by including various parameters like Serum bilirubin, Platelet count, Serum ALP, and Serum Albumin. Another non-invasive imaging modality, Fibroscan was used as the confirmatory measure [3]. There is a paucity of literature on the applicability of NFI in predicting liver fibrosis. Therefore, this study was carried out to validate and compare NFI with other available non-invasive serum indices.

In this study, the age of patients ranged from 18 to 82 years. The mean age of patients was 45±14.25 years which was in concordance with the study conducted by Hussain AR et al [3] in which 1898 patients were enrolled and the mean age of the patients was 41.5 years. In our study, 64.8% of patients were males which was in concordance with the study conducted by Huang R et al [10] in which 60.8% of patients were males. In the present study, hepatitis virus infection was the most common etiology (47%) (Hepatitis B virus-related 29.3%, Hepatitis C virus-related 23.3%) followed by alcoholism (22%). There were only 31% of cases affected by other etiologies out of which 29% of etiologies were being still investigated. The distribution of Chronic liver disease patients according to etiology was in concordance with the multi-centric epidemiological study from India [11].

Platelet count too ranged from as low as 0.20 lakhs/mm^3^ to 4.5 lakhs thousand/mm^3^ with a mean value of 1.1 ± 0.7 lakhs/mm^3^. Thrombocytopenia in patients with Chronic Liver Disease is explained by portal hypertension leading to pooling of platelets in an enlarged spleen or reduced hepatic production of thrombopoietin. The inverse correlation of platelet count and the degree of hepatic fibrosis in chronic liver disease has been noted by several investigators [12,13].

In our study, the mean Serum ALP was 327 ± 215 IU/L which was concordant with the study done by Hussain AR et al [3] in which the mean Serum ALP was 302±138 IU/L.

The mean serum total bilirubin level in our study was 2.8 ± 4.1mg/dl. The mean total bilirubin level in our study was 1.07 ± 0.81 in F0-F3 stage patients and 3.2 ± 4.49 in F4 stage patients which was concordant with the study done by Ahmad W et al [14], in which there was a gradual increase in total serum bilirubin levels in fibrosis stages.

The mean serum albumin levels in patients in our study were 3.0 ± 0.7 g/dl which was in concordance with the study conducted by Hussain AR et al [3] in which the mean serum albumin level was 3.4 ± 1.2 g/dl. The findings were in concordance with the study done by Carvalho JR et al [15] in which advanced cirrhosis is associated with a decrease in plasma albumin.

In our study, the most common fibrosis stage among the patients enrolled was the F4 stage (77.4%) followed by the F0-F1 stage (12%) . The distribution of patients was in discordance with the study done by Hussain AR et al [3] in which the maximum number of patients were in F0-F1 (54.5%).

Correlation of various non-invasive serum indices for liver fibrosis with liver stiffness score by Fibroscan was done and all the indices included in the study had a positive correlation but a significant correlation (p-value <0.05) was found only with AAR (p-value 0.004), FCI (p-value 0.008), FI (p-value 0.013), NFI (p-value 0.033).

For the F4 stage of liver fibrosis, the optimum cut-off of NFI in our study was ≥ 6670 with a sensitivity of 75.8% and specificity of 81.8%, and the AUC was 0.848, which was in concordance with the study done by Hussain AR et al [3], in which the optimum cut off for F4 stage for NFI was >30.94 with a specificity of 72.1% and sensitivity of 47.1% with an AUC of 0.831. NFI was found to have a good performance (AUROC 0.80-0.90) to differentiate the F4 stage (cirrhosis) from the F0-F3 stage in our study, but the cutoff value for the F4 stage in our study was high as compared to the study done by Hussain AR et al [3] due to variation in the distribution of patients in various fibrosis stages. The majority of the patients in our study were in the F4 stage whereas in the study done by Hussain AR et al [3], the majority of the patients were in the F0-F1 stage. NFI had the highest area under the curve for the F4 stage compared to other indices like FCI (0.836), FI (0.747), AAR (0.665), FIB4 (0.662), APRI (0.618), Pohl Score (0.585) and API (0.580).

In our study, NFI had a cutoff value of ≥ 2112 for the F3 stage of liver fibrosis with a sensitivity of 63.6% and specificity of 72.7% with AUC 0.669 which was in concordance with the study conducted by Hussain AR et al [3] in which NFI had a cutoff of >11.64 with a sensitivity of 61% and specificity of >75% with AUC 0.609 indicating poor performance in differentiating F3 stage from F0-F2 stage of liver fibrosis. The discordance in cutoff values for NFI in both studies for the F3 stage was due to the difference in the distribution of patients. For F3 stage, NFI had the highest AUC compared to APRI (0.660), AAR (0.636), FI (0.616), FIB4 (0.603), FCI (0.603), and API (0.526).

The optimum cutoff of NFI in our study for the F2 stage of liver fibrosis was ≥1334 with a sensitivity of 100% and specificity of 56.3% with AUC 0.688 having poor performance in differentiating the F2 stage from the F0-F1 stage. All other indices had AUC ≤ 0.500 and failed to predict the F2 stage.

Limitations

Our study enrolled patients from a single tertiary care center. The sample size of the study was small. There was a non-uniform distribution of patients in different stages of liver fibrosis with the majority of patients having F4 fibrosis. The reason for non-uniform distribution is that most patients referred to a tertiary care center are in advanced stages of fibrosis. Liver stiffness measurement by transient elastography was taken as reference for comparing various serum indices in determining stage of liver fibrosis, though liver biopsy could give more accurate measurements of fibrosis. NFI was initially introduced by Hussain AR et al [3] for assessing fibrosis in patients of chronic hepatitis C virus infection, so the findings may not be generalizable to chronic liver diseases from other etiologies.

## Conclusions

NFI had the highest performance in differentiating all the fibrosis stages in patients of chronic liver disease when compared to other non-invasive serum indices for liver fibrosis like AAR, APRI, FIB-4, FCI, FI, API, and Pohl score included in our study for various fibrosis stages as suggested by AUROC values. However, all the non-invasive serum indices including NFI had poor performance or failed to differentiate the F3, F2, and F0-F1 stages in our study. In our study, most of the patients were in an advanced stage of liver fibrosis. To overcome this, a multicentric study can be performed. Further studies on a larger population to validate the findings of the present study are recommended.
